# Godey’s Lady’s Book for 1864

**Published:** 1864-01

**Authors:** 


GODEY'S LADY'S BOOK FOR 1864.

We are in receipt of the January number of this popular Magazine and observe that for 1864, great improvements are proposed by the publishers. For the last thirty-four years this Magazine has been one of the most popular literary publications of the country. The illustrations, fashion-plates, and engravings, are always in the highest style of the art, while the communications are by the most eminent and popular authors. The terms are as usual, $3 per year; Two copies, $5; Three copies, $6; Four copies, $7. Club Terms still more favorable; for details of which, notice January uumber.

